# Potential effect of FLASH radiotherapy on testicular damage: a review of current evidence

**DOI:** 10.3389/fonc.2025.1618270

**Published:** 2025-09-25

**Authors:** Xiaoyu Zhi, Lehui Du, YaNan Han, Weiwei Li, Pei Zhang, Xingdong Guo, Yuan Wang, Na Ma, Xiao Lei, Baolin Qu

**Affiliations:** ^1^ The First Medical Center of the Chinese People’s Liberation Army (PLA) General Hospital, Beijing, China; ^2^ Medical School of the Chinese PLA, Beijing, China; ^3^ The 81st Group Army Hospital of the Chinese PLA, Zhangjiakou, China

**Keywords:** FLASH irradiation, ultra-high dose rate, radiation damage, testicular tissue, reproductive cells

## Abstract

Radiotherapy is a major source of ionizing radiation that adversely affects the male reproductive system. FLASH radiotherapy (FLASH-RT), a novel technique delivering ultra-high dose rate (UHDR) radiation, has shown promise in reducing normal tissue damage while maintaining antitumor efficacy—a phenomenon known as the “FLASH effect”. Male fertility depends on the coordinated function of spermatogenic, Sertoli, and Leydig cells in the testes, which display differential sensitivity to radiation exposure. Due to ethical limitations in human studies, rodent models are indispensable for exploring radiation-induced testicular injury. This review summarizes current evidence of the FLASH effect across rodent organs and the impact of ionizing radiation at different dose rates on testicular cells. Given the current lack of direct evidence for the FLASH effect in testicular tissue, it also reviews mechanisms observed in other organs that may contribute to its potential protective role in the testes. A deeper understanding of these mechanisms could inform fertility-preserving strategies in male cancer patients receiving radiotherapy.

## 1 Background

Radiotherapy is a key treatment modality for various cancers. It has achieved remarkable success in the long-term control of diseases such as Hodgkin’s lymphoma (1), rectal cancer (2), and prostate cancer (3). However, the treatment areas for these cancers are often located near the male reproductive system, and radiation exposure could cause damage to reproductive organs (4–6). This may impair spermatogenesis, disrupt hormonal secretion, and even result in infertility. Currently, sperm cryopreservation is the most effective fertility preservation strategy for adult male patients undergoing radiotherapy (7). In contrast, for prepubertal boys, the only feasible option for preserving fertility lies in the protection of endogenous germ cells (8). With rising survival rates among cancer patients, maintaining quality of life after treatment—particularly preserving reproductive health—has become a critical focus in clinical management.

In 2014, Favaudon et al. (9) at the Curie Institute in France demonstrated a pronounced differential response between tumor and normal tissues when irradiated under ultra-high dose rate (UHDR) conditions. This was the first work to define this technique as FLASH radiotherapy (FLASH-RT). Compared to conventional radiotherapy (CONV-RT), FLASH-RT (delivered at average dose rates exceeding 40 Gy/s within less than 200 ms) significantly suppressed tumor growth in mouse models of lung cancer while markedly reducing adverse effects in surrounding normal tissues (9). The observation that normal tissue damage is reduced without compromising tumor control has been termed the “FLASH effect” (10). Building on encouraging preclinical results, a prospective, non-randomized clinical study at the Cincinnati Children’s Hospital/UC Health Proton Therapy Center investigated FLASH-RT in patients with bone metastases (11, 12). The findings demonstrated the clinical feasibility of FLASH-RT, with treatment efficacy and toxicity profiles comparable to those of CONV-RT. The emergence of FLASH-RT offers a new strategy to reduce normal tissue damage during radiotherapy and holds promise for making significant breakthroughs in future clinical practice.

The testes serve as both the site of sperm production (through spermatogenesis) and the primary source of male sex hormones (via steroidogenesis), both of which are essential for maintaining normal reproductive function in adult males. The major cellular components of the testes include germ cells, Sertoli cells, and Leydig cells (13). These cells exhibit significant differences in their sensitivity to ionizing radiation. Spermatogonia are highly susceptible to radiation, and their damage can directly impair fertility (14). Although Sertoli and Leydig cells are relatively resistant to radiation, impairment of their functions can still indirectly disrupt the spermatogenic process (15). Therefore, whether FLASH-RT causes less testicular damage than CONV-RT remains an important question and warrants further investigation.

This review will provide a comprehensive summary of current studies investigating the FLASH effect across various rodent organs. We will analyze the pivotal roles of spermatogonia, Sertoli cells, and Leydig cells in male reproduction and review their responses to ionizing radiation at varying dose rates. Finally, potential mechanisms underlying the FLASH effect in the testes are discussed. Through a synthesis of current evidence, this review aims to offer new insights into optimizing radiotherapy protocols, minimizing reproductive toxicity, and advancing the clinical translation of FLASH technology.

## 2 Studies on the FLASH effect in rodent organs

**Figure 1:**
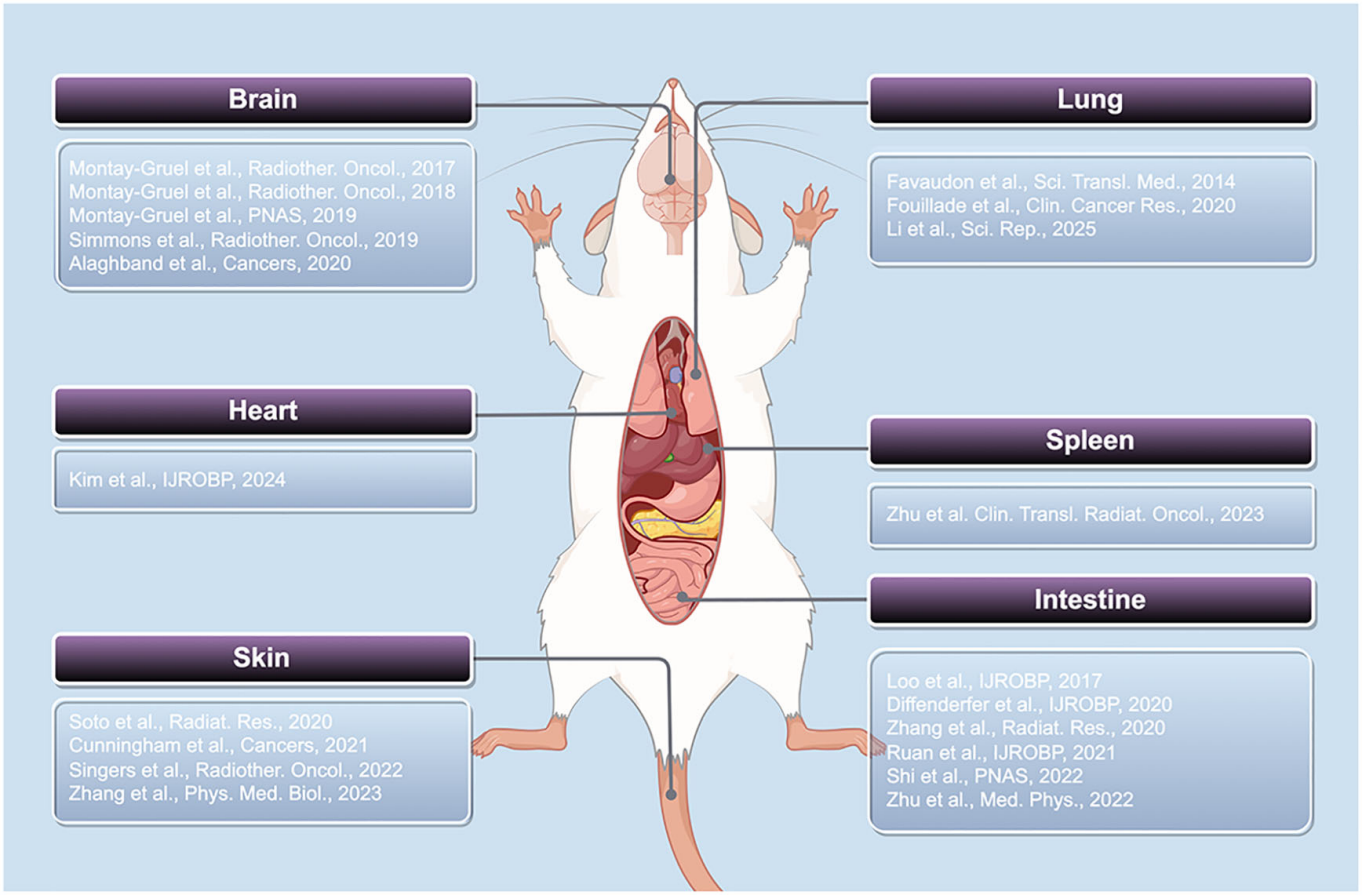
Studies on the FLASH effect in rodent organs.

In this section, we summarize recent research progress on the FLASH effect across various organs in rodent models, focusing on acute and chronic skin injury, cognitive impairment associated with the nervous system, cardiac dysfunction, pulmonary fibrosis, intestinal crypt damage, and immunological injury in the spleen. These findings highlight distinct tissue responses under FLASH-RT compared to CONV-RT, supporting the notion that the FLASH effect occurs across a range of organ systems. These findings not only provide experimental support for the clinical application of FLASH-RT but also lay the groundwork for elucidating its protective mechanisms against radiation-induced testicular injury (**Figure 1**).

### 2.1 Skin

The research team led by Soto (16) was the first to investigate the effects of electron FLASH-RT (average dose rate: 180 Gy/s) versus CONV-RT (average dose rate: 0.0747 Gy/s) on skin toxicity in mice. Their study demonstrated that, eight weeks after a single high-dose 40 Gy hemithoracic irradiation, FLASH-RT significantly reduced both the incidence and severity of skin ulceration (*p* < 0.05). Furthermore, survival rates were markedly higher in mice treated with FLASH-RT compared to those receiving CONV-RT. Specifically, in the FLASH-RT group, median survival exceeded 180 days for both the 30 Gy and 40 Gy doses, whereas in the CONV-RT group, it was 100 days and 52 days, respectively. Subsequently, Singers’ team (17) investigated the effects of proton FLASH-RT on normal skin injury. By generating a complete dose–response curve for acute skin damage grading, they found that, under conditions producing equivalent biological effects, FLASH-RT (average dose rate: 65–92 Gy/s) required doses that were 44–58% higher than those used in CONV-RT (average dose rate: 0.35–0.40 Gy/s). Further work by Zhang et al. (18) involved single-dose proton beam irradiation of mouse skin at total doses of 25 Gy, 27 Gy, 30 Gy, and 45 Gy. At doses ranging from 25 to 30 Gy, FLASH-RT (average dose rate: 130 Gy/s) reduced skin contraction by approximately 15% compared to CONV-RT (average dose rate: 0.4 Gy/s). At 75 days post-irradiation, measurements of epidermal thickness and collagen deposition indicated that FLASH-RT induced less skin damage. Interestingly, the dose rate–dependent protective effect of FLASH-RT was abolished when mice were either hyperoxygenated or when skin oxygen concentrations were reduced. Cunningham’s team (19) provided additional evidence that proton FLASH-RT at a total dose of 35 Gy (average dose rates: 57 Gy/s and 115 Gy/s) resulted in significantly lower acute skin toxicity in mice compared to CONV-RT (average dose rate: 1 Gy/s). Moreover, mice in the FLASH-RT group showed markedly reduced levels of transforming growth factor-β1 (TGF-β1) in both plasma and skin. In addition, late radiation-induced effects, such as hind limb contracture, were significantly alleviated in these mice. Taken together, these studies suggest that the FLASH effect is associated with the mitigation of radiation-induced acute and chronic skin damage.

### 2.2 Brain

In 2017, Montay-Gruel and colleagues (20) were the first to report the effects of whole-brain irradiation (WBI) using electron beam FLASH-RT (average dose rate >100 Gy/s) in adult mice. Their study demonstrated that a single 10 Gy dose delivered at FLASH dose rates significantly reduced radiation-induced toxicity in normal brain tissue, compared to conventional dose rates (average dose rate: 0.1 Gy/s), particularly by alleviating damage to hippocampal cell proliferation and preserving spatial memory performance. The team further investigated the mechanisms underlying the reduced brain toxicity observed with FLASH-RT (instantaneous dose rate ≥1.8 × 10^6^ Gy/s). They found that elevating brain oxygen tension by inhaling a carbon monoxide–oxygen gas mixture could abolish the FLASH effect. Radiochemical analyses revealed lower levels of toxic reactive oxygen species (ROS, such as hydrogen peroxide) following FLASH-RT exposure. These findings suggest that FLASH-RT maintains neuronal morphology and dendritic spine density by mitigating oxidative stress-dependent neuroinflammation (21). Simmons and colleagues (22) further confirmed the neuroprotective role of the FLASH effect. Ten weeks after 30 Gy of whole-brain electron irradiation, mice treated with FLASH-RT (average dose rate: 200–300 Gy/s) showed significantly less impairment in novel object location tasks (*p* =0.049). These mice also exhibited reduced dendritic spine loss in the hippocampus and fewer CD68-positive microglia, compared to those receiving CONV-RT (average dose rate: 0.13 Gy/s). Moreover, CONV-RT significantly upregulated five of ten pro-inflammatory cytokines (IL-6, IL-1β, TNFα, KC/GRO, and IL-4) in the hippocampus, while FLASH-RT only mildly elevated three (IL-1β, TNFα, KC/GRO). Alaghband’s team (23) further investigated the FLASH effect observed in the brain tissue of radiosensitive juvenile mice. In their study, WBI was performed using electron FLASH-RT with a total dose of 8 Gy at an instantaneous dose rate ≥4.4 × 10^6^ Gy/s. The study found that, compared to CONV-RT (average dose rate: 0.077 Gy/s), FLASH-RT resulted in milder cognitive deficits, potentially due to the FLASH effect contributing to the protection of both developing and mature neurons, a reduction in microglial proliferation, and a mitigation of the decline in plasma growth hormone (GH) levels. Montay-Gruel’s group (24) also conducted WBI experiments using X-rays. Mice were irradiated with a 10 Gy dose delivered at an average dose rate of 37 Gy/s. Over a six-month follow-up, FLASH-RT–treated mice exhibited no signs of radiation-induced memory loss, in contrast to those treated with CONV-RT (average dose rate:0.05 Gy/s). The reduced brain toxicity observed with FLASH-RT was attributed to decreased hippocampal proliferative damage and astrocytic gliosis. Across both adult and juvenile rodent models—and regardless of radiation modality (electron beams or X-rays)—FLASH-RT consistently reduced radiation-induced neurotoxicity compared to CONV-RT, highlighting its promise as a strategy to minimize adverse effects on the brain during radiotherapy.

### 2.3 Heart

Kim and colleagues (25) explored the cardioprotective mechanisms associated with the FLASH effect by delivering a single 40 Gy dose of proton beam irradiation to the apical region of the mouse heart. The dose rate was 0.84 ± 0.07 Gy/s in the CONV-RT group and 122.65 ± 2.35 Gy/s in the FLASH-RT group. Two weeks after irradiation, RNA sequencing was performed to evaluate gene expression changes, and immunofluorescence staining was conducted to assess TGF-β1 levels. CONV-RT notably upregulated multiple pathways associated with the DNA damage response, activation of the tumor necrosis factor (TNF) superfamily, and inflammatory signaling. In contrast, FLASH-RT primarily modulated pathways related to cytoplasmic translation, mitochondrial organization, and adenosine triphosphate (ATP) synthesis. TGF-β1 levels were significantly elevated in the CONV-RT group, while levels in the FLASH group were comparable to the control (*p* < 0.01). At three weeks post-irradiation, cardiac inflammatory responses were assessed using multiplex cytokine assays and immunofluorescence. The FLASH group exhibited significantly lower levels of the pro-inflammatory cytokine TNF-α compared to the conventional group (*p* < 0.05). Although the difference in interferon-gamma (IFN-γ) levels was not statistically significant, IFN-γ levels were also lower in the FLASH group, suggesting a distinct dynamic profile of cytokine responses between the two irradiation modalities. At 30 weeks post-irradiation, perivascular fibrosis was quantitatively assessed using Masson’s trichrome staining and Picrosirius red staining. Quantitative analysis showed significantly reduced vascular wall thickening and collagen deposition in the FLASH group compared to the CONV-RT group (*p* < 0.05), with values similar to those of the control. Similarly, Picrosirius red staining demonstrated that collagen deposition around blood vessels was significantly lower in the FLASH group than in the CONV-RT group (*p* < 0.001). These findings suggest that FLASH-RT mitigates radiation-induced myocardial fibrosis compared to CONV-RT, potentially by limiting the development of a chronic inflammatory environment following irradiation. Echocardiography at 8- and 30-weeks post-irradiation confirmed that FLASH-RT caused less functional cardiac impairment than CONV-RT.

### 2.4 Lung

Favaudon and colleagues (9) investigated pulmonary fibrosis development in mice after a single exposure to electron beam FLASH-RT (average dose rate ≥40 Gy/s) compared to CONV-RT (average dose rate ≤0.03 Gy/s). Their results demonstrated that CONV-RT at a dose of 15 Gy induced significant pulmonary fibrosis, which was associated with activation of the TGF-β signaling cascade. In contrast, FLASH-RT administered at total doses below 20 Gy did not lead to any detectable pulmonary complications for at least 36 weeks post-irradiation. Importantly, FLASH-RT significantly reduced radiation-induced apoptosis in normal smooth muscle and epithelial cells compared to CONV-RT. Mechanistic investigations further suggested that the mitigation of pulmonary injury by FLASH-RT might be attributed to decreased DNA damage and reduced telomerase-associated injury, thereby suppressing cellular senescence-related signaling pathways (26). Li and colleagues (27) established a lung injury model by exposing mice to total-body irradiation with electron beams at a single dose of 3 Gy (average dose rate: 200 Gy/s for FLASH and 0.3 Gy/s for CONV). Results showed that mice in the FLASH-RT group exhibited less lung tissue damage and lower levels of fibrosis compared to the CONV-RT group. Proteomic analysis revealed a marked difference in the expression of the CCT6b protein between the two irradiated groups (*p* < 0.01). Subsequent Western blot and immunofluorescence analyses confirmed that CCT6b expression was significantly lower in the conventional group than in the FLASH group. Moreover, downregulation of CCT6b was associated with a substantial reduction in E-cadherin expression and a concurrent upregulation of α-smooth muscle actin (α-SMA) and vimentin. These findings indicate that, compared to CONV-RT, FLASH-RT offers more significant advantages in reducing radiation-induced damage to normal lung tissue.

### 2.5 Intestine

In 2017, Loo and colleagues (28) from Stanford University conducted a comprehensive analysis of data from 178 mice subjected to total abdominal electron beam irradiation. They found that the LD50 for conventional irradiation (average dose rate: 0.05 Gy/s) was 14.7 Gy, whereas the LD50 for FLASH irradiation was 17.5 Gy (with average dose rates of 70 Gy/s and 210 Gy/s yielding LD50 values of 16.6 Gy and 18.3 Gy, respectively). Building upon this, Diffenderfer and colleagues (29) studied acute intestinal injury following 15 Gy of total abdominal electron irradiation. They observed that the number of proliferating cells in the intestinal crypts of mice in the FLASH-RT group (average dose rate: 78 ± 9 Gy/s) was significantly higher than in the conventional group (average dose rate: 0.9 ± 0.08 Gy/s; *p* = 0.0016). At 8 weeks following 18 Gy irradiation to the small intestine, the conventional group exhibited pronounced intestinal fibrosis, whereas the FLASH group displayed significantly reduced fibrotic changes (*p* = 0.0014), with histological features closely resembling those of non-irradiated controls. Further insights from Ruan et al. (30) demonstrated that electron beam FLASH-RT (instantaneous dose rates: 2–6 × 10^6^ Gy/s) alleviated acute crypt injury in the small intestine by reducing alterations in gut microbiota composition. In 2020, Zhang and colleagues (31) at Harvard Medical School irradiated the abdomens of mice with a total proton dose of 16 Gy. They found that the FLASH-RT group (average dose rate >100 Gy/s) had significantly higher survival rates compared to the CONV-RT group (average dose rate 1.9–4.5 Gy/min; *p* = 0.049). Histological analysis indicated improved late-phase repair in the submucosal and muscular layers of the small intestine in the FLASH group. Shi and colleagues (32) further proposed that X-ray FLASH-RT (average dose rate: 110–120 Gy/s) mitigates intestinal injury in mice by significantly suppressing the cGAS–STING signaling pathway through reduction of cytosolic double-stranded DNA levels, thereby reducing the proportion of CD8^+^ T lymphocytes in intestinal crypts from 22.7% in the CONV-RT group to 11.9%. In a six-week follow-up study, Zhu et al. (33) compared the effects of 15 Gy X-ray irradiation between FLASH (average dose rate >150 Gy/s) and conventional groups. Mice in the FLASH group demonstrated better recovery in body weight (*p* < 0.05) and higher survival rates. Histological evaluation revealed that acute intestinal injury was notably milder in the FLASH group. During the acute phase, both groups exhibited increased numbers of inflammatory blood cells and elevated levels of pro-inflammatory cytokines. However, during the late phase, the FLASH group showed significantly reduced infiltration of inflammatory cells (including white blood cells and lymphocytes) and lower concentrations of cytokines such as TNF-α, IL-6, and IL-10. It is noteworthy that, despite elevated ROS signaling during the acute phase in the FLASH group, lipid peroxidation levels remained lower than those observed in the conventional group, indicating a potential advantage of FLASH-RT in mitigating oxidative stress.

### 2.6 Spleen

Zhu and colleagues (34) exposed mice to 10 Gy of abdominal X-ray irradiation and assessed them four weeks post-exposure. Their results demonstrated that spleen weights in the FLASH group (average dose rate: 125 Gy/s) were significantly lower than those in both the control and CONV-RT groups (average dose rate: 0.2 Gy/s; *p* < 0.05). Flow cytometry analysis revealed a markedly higher CD8^+^/CD3^+^ ratio (*p* < 0.01) and a significantly lower CD4^+^/CD3^+^ ratio (*p* < 0.01) in the spleens of FLASH-irradiated mice compared to both control and CONV-RT mice, suggesting that FLASH-RT may enhance systemic immune activation. H&E staining revealed prominent red pulp expansion in the spleens of CONV-RT–treated mice, whereas such changes were absent in the FLASH group.

## 3 Testicular structure: spermatogonia, Sertoli cells, and Leydig cells

**Figure 2:**
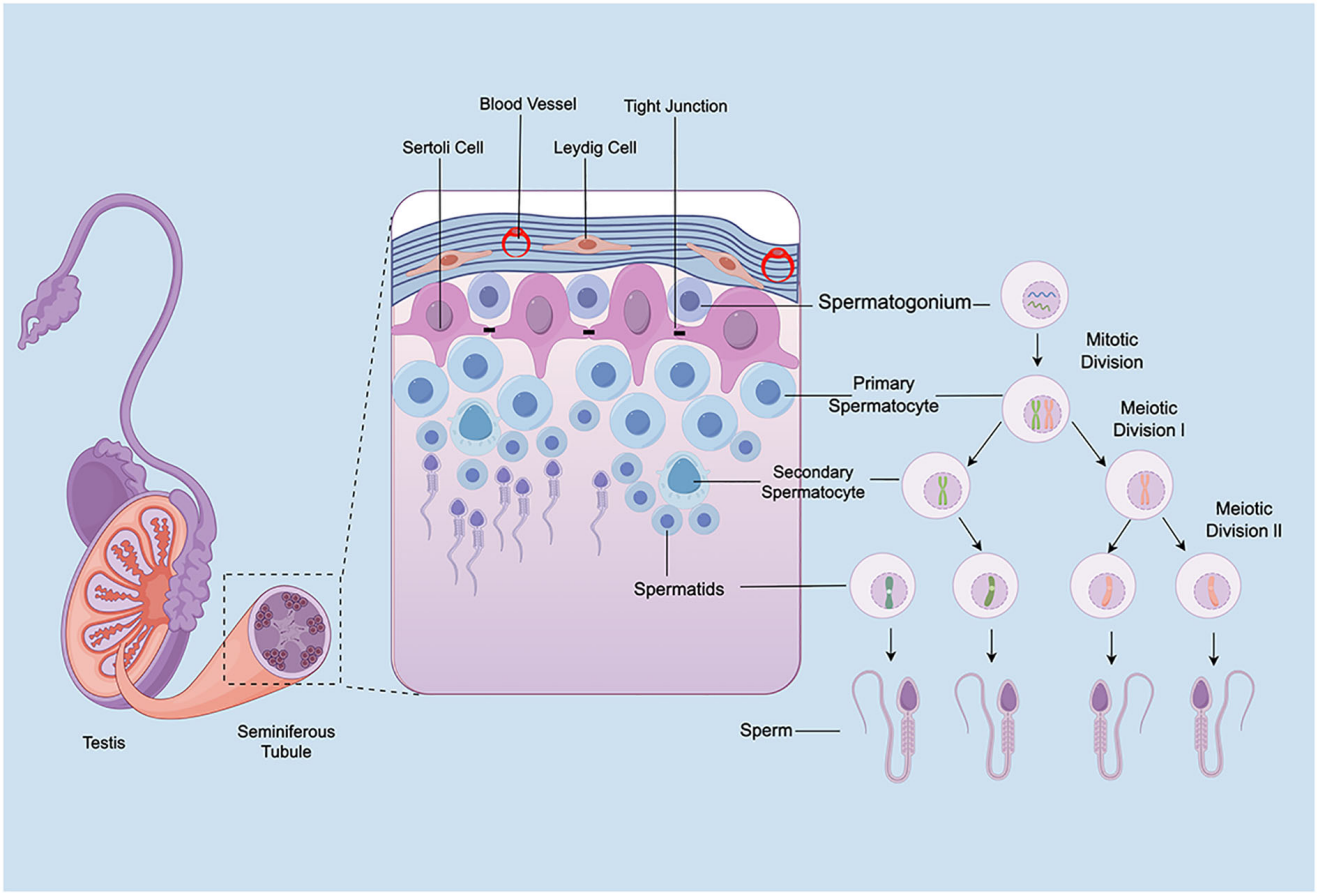
Schematic of testicular structure and spermatogenesis.

Anatomically, the testes consist of two primary compartments: the interstitial tissue and the seminiferous tubules. This section highlights the structural characteristics of rodent testes and discusses the respective roles of spermatogonia, Sertoli cells, and Leydig cells in spermatogenesis, thereby underscoring the importance of mitigating radiotherapy-induced testicular damage (**Figure 2**).

### 3.1 Spermatogonia

Spermatogonia, located adjacent to the basement membrane of the seminiferous tubules, represent the foundational population for spermatogenesis. In rodents, the differentiation of spermatogonia is a tightly regulated and intricate process. Stem cells (As) self-renew to maintain the stem cell pool while giving rise to paired undifferentiated A-type spermatogonia (Apr), which remain connected via cytoplasmic bridges. These further divide into aligned chains (Aal) that sequentially progress through six mitotic divisions into A1–A4, intermediate (In), and B-type spermatogonia. The latter giving rise to primary spermatocytes (35). These primary spermatocytes undergo the first meiotic division to form secondary spermatocytes, which immediately proceed to the second meiotic division to generate spermatids (13). Spermatids then undergo spermiogenesis, a process involving significant morphological changes including acrosome formation, flagellum development, nuclear elongation, and cytoplasmic reduction, ultimately resulting in mature spermatozoa (36). The entire process is intricately regulated by the hypothalamic–pituitary–gonadal (HPG) axis. Gonadotropin-releasing hormone (GnRH) from the hypothalamus stimulates the anterior pituitary to secrete follicle-stimulating hormone (FSH) and luteinizing hormone (LH), both essential for spermatogonial proliferation and differentiation (37). Notably, spermatogonia exhibit high sensitivity to ionizing radiation, with even low doses capable of causing severe impairments in spermatogenesis (38). Understanding the mechanisms by which ionizing radiation affects spermatogonia is essential for developing protective strategies and improving post-radiotherapy fertility outcomes.

### 3.2 Sertoli cells

Sertoli cells play a crucial role in male reproductive physiology. Residing within the seminiferous tubules, they are the only somatic cell type present in the seminiferous epithelium (39), originally described by Enrico Sertoli in 1865 in *Il Morgagni (* 40). These cells undergo significant morphological and functional maturation from the fetal period to puberty, transitioning from proliferative and immature cells into non-proliferative, functionally competent cells capable of supporting spermatogenesis (41). Mature Sertoli cells exhibit a columnar morphology, with their basal ends anchored to the basement membrane and apical projections extending into the tubule lumen. Their cytoplasm is metabolically active, rich in rough endoplasmic reticulum, Golgi apparatus, and mitochondria (42). Structurally, Sertoli cells are connected by tight junctions, gap junctions, and desmosomes, contributing to the formation of the blood–testis barrier, which partitions the seminiferous epithelium into basal and adluminal compartments (43). The number of Sertoli cells strongly correlates with testes weight and sperm count (44). In addition to structural support, Sertoli cells establish an immunoprivileged environment and supply essential nutrients to germ cells (45). Each Sertoli cell supports approximately 30 to 50 germ cells at various developmental stages (46). Their metabolic byproducts, such as lactate and pyruvate, serve as crucial energy sources for germ cells (47, 48). During spermatogenesis, Sertoli cells actively remodel their intercellular junctions through the secretion of proteases and signaling molecules, facilitating germ cell translocation across the epithelium (49). Their function is tightly regulated by FSH and testosterone (50). FSH stimulates Sertoli cells to synthesize androgen-binding protein (ABP), which facilitates the accumulation of androgens within the seminiferous epithelium, promoting their binding to intracellular androgen receptors and thereby enhancing the effects of androgens on spermatogenesis (51). Meanwhile, androgens act on Sertoli cells to increase their responsiveness to FSH, further promoting the secretion of ABP and other nutritive factors essential for spermatogenesis (52). Therefore, ABP serves as a key biochemical marker of Sertoli cell function and blood–testis barrier integrity (53). In summary, Sertoli cells are indispensable for male reproductive function, contributing to structural support, immunological protection, nutrient provision, and hormonal regulation. Therefore, in-depth investigations into Sertoli cell biology are essential for understanding male reproductive physiology, particularly in the context of evaluating the effects of radiotherapy on fertility preservation.

### 3.3 Leydig cells

Leydig cells, first identified by Franz Leydig in 1850, reside in the interstitial space between seminiferous tubules and represent the principal source of testosterone synthesis and secretion (54). Testosterone is essential for multiple aspects of male reproductive health, including testicular development, sexual differentiation, spermatogenesis, and fertility (55–57). In rodents, Leydig cells are classified into two types: fetal Leydig cells (FLCs) and adult Leydig cells (ALCs). FLCs are round or oval-shaped, contain abundant lipid droplets, are typically clustered, and are enclosed by a basement membrane composed of collagen and laminin. In contrast, ALCs are larger, contain dense, round nuclei, often form aggregates, and are not surrounded by a basement membrane. FLCs are primarily responsible for testosterone production during fetal development (58). In rats, FLCs begin testosterone synthesis around embryonic days 15.5–16.5 (59). In mice, FLCs synthesize androstenedione, which is subsequently converted into testosterone by fetal Sertoli cells (60, 61). Genetic studies in mice have shown that the maintenance and differentiation of FLCs depend on a delicate balance between pro-differentiation and anti-differentiation signals (62). ALCs emerge during puberty, originating from undifferentiated mesenchymal-like stem cells in the testicular interstitium. Their development proceeds through several stages: stem Leydig cells, progenitor Leydig cells, immature Leydig cells, and ultimately, mature ALCs (63). As FLC numbers decline postnatally, testosterone levels decrease and reach a nadir, followed by a gradual increase as ALCs develop and testosterone production rises to adult levels (64). The function of ALCs is regulated by multiple hormones, primarily LH and FSH (65, 66). Disruption of Leydig cell function is often associated with impaired spermatogenesis (67). In summary, Leydig cells are essential for the male reproductive system. Their primary role is the synthesis and secretion of testosterone, which is indispensable for male sexual development and reproductive capacity.

## 4 Differential effects of radiation dose rates on spermatogonia and Sertoli cells

Current studies indicate that the effects of radiation on different types of testicular cells largely depend on dose rate, although most of these studies have not reached the average dose rate threshold required for FLASH-RT (>40 Gy/s). Findings concerning spermatogonia and Sertoli cells suggest that changes in dose rate can significantly influence their survival and function. To date, no studies have specifically investigated the effects of different dose rates on Leydig cell function. Given the current knowledge gap, investigating dose-rate–dependent effects on Leydig cells should be a priority for future research. Therefore, this review focuses on the effects of varying radiation dose rates on spermatogonia and Sertoli cells, aiming to inform future studies on the impact and mechanisms of FLASH-RT in testicular tissue. These findings are summarized in **Table 1** for clarity.

**Table 1:** Summary of studies on the effects of different radiation dose rates on the testes, spermatogonia, and Sertoli cells.

Team	Study period	Study subjects	Radiation type	Dose rates	Doses	Results
Watanabe’s Team (68)	2017	testes	γ-rays	659−690 mGy/min 0.303 mGy/min	2Gy	Prenatal exposure to two different radiation dose rates resulted in distinct effects on testicular weight and the number of functional seminiferous tubules in adult mice.
Delic’s Team (69)	1987	spermatogonia	γ-rays	1.1cGy/min 11 cGy/min 112 cGy/min	8-20Gy	As the radiation dose rate decreases, the survival rate of stem spermatogonia increases significantly.
Bae’s Team (70)	2021	spermatogonial stem cells meiotic cells	γ-rays	3.4 mGy/h 51 Gy/h	8Gy	Low-dose-rate radiation exacerbates damage to spermatogonia and meiotic germ cells.
Pinon-Lataillade’s Team (71)	1998	Sertoli cells	γ-rays	7 cGy/day 3 Gy/min	9.1 Gy 9Gy	Under comparable total irradiation doses, radiation at different dose rates affects Sertoli cell function at distinct time points.

### 4.1 Spermatogonia

Watanabe and colleagues (68) evaluated dose rate-dependent effects of radiation exposure during different developmental stages (fetal, neonatal, and juvenile periods) on spermatogenesis in adulthood (postnatal week 10) in mice. Mice were exposed to 2 Gy of γ-radiation delivered either acutely (659–690 mGy/min) or chronically (0.303 mGy/min) conditions. Acute irradiation on gestational days 15.5–17.5 markedly impaired testicular development, reducing testes weight to ~20% of control levels. When irradiation occurred on gestational days 18.5 or 19.5, testes weight was approximately 60% of controls. In contrast, chronic irradiation from gestational day 14.5 to 19.5 reduced testes weight to 33.4% of controls. Further analysis revealed that the effective seminiferous tubule ratios in adult mice exposed acutely on gestational days 15.5, 16.5, 17.5, 18.5, and 19.5 were approximately 13.8%, 2.9%, 13.4%, 93.1%, and 91.4%, respectively. For chronically irradiated mice, the effective tubule participation rate in adulthood was 44.3%, and epididymal sperm counts were sufficient for successful *in vitro* fertilization. Importantly, neither acute nor chronic irradiation during the postnatal period adversely affected testicular development. These findings suggest that radiation dose rate has differential effects on testes weight and the proportion of functional seminiferous tubules. Delic and colleagues (69) further investigated the impact of different radiation dose rates on germ cell survival in mice. In their study, testicular tissue was analyzed 35 days after exposure to 8–20 Gy of γ-radiation delivered at dose rates of 1.1, 11, and 112 cGy/min, focusing on clonogenic stem spermatogonia survival. Additionally, sperm head counts were evaluated 29 days after 3 Gy exposure at dose rates of 2.5, 5, 11, and 112 cGy/min to assess the survival of differentiating spermatogonia. Results showed that as the dose rate decreased, the survival of stem spermatogonia significantly increased, with the Do value of the survival curve rising from 2.86 ± 0.29 Gy at 112 cGy/min to 4.46 ± 0.45 Gy at 1.1 cGy/min. However, the survival of differentiating spermatogonia was not significantly affected by dose rate. In contrast, Bae et al. (70)reported that low-dose-rate exposure induced even more severe testicular toxicity, highlighting the complexity of dose-rate effects. Mice were exposed to a total dose of 8 Gy γ-radiation at either a low dose rate (~3.4 mGy/h) or a high dose rate (~51 Gy/h). Compared to high-dose-rate exposure, low-dose-rate radiation significantly reduced the expression of spermatogonial stem cell markers such as Plzf, c-Kit, and Oct4. Furthermore, fluorescence-activated cell sorting analysis showed that, except for round spermatids, all stages of spermatogonia and meiotic cells were markedly reduced under low-dose-rate exposure. This was accompanied by negative effects on spermatogenesis, including reduced sperm count and motility, as well as increased sperm abnormalities. Although these findings may appear contradictory at first glance, it is important to note that the so-called “low” dose rate in Delic’s study (1.1 cGy/min) is actually over 300 times higher than that used in Bae’s study (~0.0057 cGy/min). Collectively, these studies underscore the critical but complex role of radiation dose rate in testicular biology. They offer valuable experimental evidence for understanding the dose rate-dependent effects of ionizing radiation on the male reproductive system.

### 4.2 Sertoli cells

Pinon-Lataillade and colleagues (71) investigated the effects of different radiation dose rates on Sertoli cell function. Adult rats were exposed to γ-radiation at an average dose rate of 7 cGy/day for 131 consecutive days, totaling a cumulative dose of 9.1 Gy. The researchers observed that as the number of late spermatids (LS) declined, vacuolization occurred in the Sertoli cell cytoplasm, followed by thickening and folding of the peritubular tissue. These morphological changes were accompanied by a decrease in ABP production and an increase in FSH levels. A significant positive correlation was found between LS counts and Sertoli cell functional parameters. In a subsequent experiment, the same group administered a total dose of 9 Gy at a much higher average dose rate of 3 Gy/min. In this case, ABP levels declined when germ cell loss reached the pachytene spermatocyte stage. As LS numbers declined, both FSH and LH levels increased (14). These two experiments, conducted under similar total doses but at markedly different dose rates, demonstrated that the timing and severity of Sertoli cell dysfunction were dose rate–dependent. These findings provide further support for the conclusion that radiation dose rate differentially affects Sertoli cell function.

## 5 Potential mechanisms supporting the FLASH effect in the testes

To date, the FLASH effect has been demonstrated across multiple organ systems in rodent models. Although the precise biochemical mechanisms underlying FLASH-RT remain incompletely understood, current studies suggest that the occurrence of the FLASH effect in normal tissues may involve multiple pathways, including oxygen depletion, altered generation of free radicals and peroxides, differences in DNA damage profiles, and attenuated radiation-induced inflammatory responses. Given that existing research has shown dose rate significantly affects damage to testicular cells such as spermatogonia and Sertoli cells, it is reasonable to hypothesize that the FLASH effect may also be present in testicular tissue. This hypothesis warrants further investigation and experimental validation, especially since several of these mechanisms have already been demonstrated in other organs.

Dewey et al. (72)first observed in 1959 that increasing radiation dose rates significantly improved the survival of *Salmonella* species. Subsequent studies confirmed this phenomenon in mammalian cells, showing that high-dose-rate irradiation could enhance cellular radioresistance, particularly under hypoxic conditions (73–75). In animal experiments, Cao and colleagues (76) further demonstrated that FLASH irradiation led to a total oxygen depletion of 2.3 ± 0.3 mmHg in normal tissues, whereas oxygen levels remained largely unchanged under CONV-RT conditions. Supporting these findings, several mathematical models have predicted rapid oxygen consumption during FLASH exposure. They emphasize that the extremely short interpulse intervals hinder effective reoxygenation via diffusion in normal tissues (77–79). Consequently, transient hypoxia has been proposed as a key mechanism underlying the reduced radiation toxicity of FLASH in normal tissues. The oxygen-depletion hypothesis has also been supported by studies in skin tissue (18). However, differences in blood perfusion and metabolic activity across tissues may lead to considerable variation in FLASH-induced oxygen depletion. For instance, the testes possess a high capillary density, which may theoretically facilitate more rapid oxygen resupply (80). Conversely, the blood–testis barrier may limit the passive diffusion of oxygen into the adluminal compartment of the seminiferous tubules (43, 81). These factors suggest a complex and potentially unique oxygenation profile in testicular tissue during FLASH exposure. However, studies investigating tissue-specific oxygen consumption dynamics under FLASH irradiation remain scarce, partly due to current technical limitations in real-time oxygen monitoring. Therefore, comprehensive studies focusing on the oxygen kinetics of testicular tissue during FLASH irradiation are essential for evaluating its reproductive protective potential and guiding future clinical applications.

The free radical hypothesis is often regarded as an extension of the oxygen depletion theory. It posits that ionizing radiation generates various ROS—such as superoxide anions (O_2_•^-^), hydrogen peroxide (H_2_O_2_), and hydroxyl radicals (•OH)—primarily through the radiolysis of water (82). These ROS are highly reactive due to their unpaired electrons and can damage essential cellular components, including DNA, proteins, and lipids, leading to oxidative stress and subsequent cell injury (83). During FLASH-RT, the UHDR rapidly depletes local oxygen, creating a transient hypoxic environment that inhibits the continued formation of ROS (84). Montay-Gruel and colleagues (21) demonstrated that FLASH-RT protects neuronal morphology and dendritic spine density by reducing oxidative stress-mediated neuroinflammation. Whether through oxygen depletion or reduced ROS generation, both hypotheses converge on the notion that FLASH irradiation induces less DNA damage compared to CONV-RT. Indeed, studies have shown that FLASH-RT significantly reduces plasmid DNA single-strand breaks (85). The testes, characterized by highly differentiated tissue and ongoing meiotic activity, are particularly sensitive to radiation-induced DNA damage (86). Thus, the ability of FLASH-RT to mitigate DNA damage may offer a promising strategy for preserving reproductive function by reducing radiotoxicity in testicular tissue.

Among the proposed mechanisms underlying the FLASH effect, the immune and inflammatory hypothesis has gained increasing attention. This hypothesis proposes that, compared to CONV-RT, FLASH-RT may induce distinct immune and inflammatory responses that not only enhance antitumor efficacy (34, 87) but also contribute to the preservation of normal tissue integrity. For instance, Shi and colleagues (32) found that FLASH irradiation reduced cytotoxic CD8^+^ T-cell infiltration into intestinal crypts, thereby alleviating tissue injury. Similarly, Zhu et al. (33)reported that mice in the FLASH group exhibited lower levels of lipid peroxidation in the intestine during the acute phase, suggesting reduced oxidative stress. Furthermore, FLASH-RT has been shown to suppress the activation of key pro-inflammatory pathways, particularly the transforming growth factor-β (TGF-β) signaling axis. This, in turn, reduces the release of inflammatory cytokines and mitigates radiation-induced damage to the skin and heart (19, 25). While these studies underscore the immunomodulatory benefits of FLASH-RT in several organs, whether similar effects extend to the testes remain unknown. Importantly, the testes are recognized as an immune-privileged organ due to its unique anatomical and physiological features (43). The immune privilege of the testes appears to involve multiple layers of immune regulatory mechanisms, including conventional immune tolerance, antigen sequestration behind the blood–testis barrier, reduced immune activation, localized immunosuppression, and antigen-specific immunoregulation. Central to these regulatory processes are the somatic cells of the testes, particularly the Sertoli cells, as well as testicular secretions such as androgens, cytokines, peptides, and bioactive lipids (88). Given the complexity of immune regulation in the testicular environment, future studies should investigate whether FLASH irradiation modulates the expression or activity of inflammatory mediators in a testes-specific manner. A better understanding of these testes-specific immune responses may reveal novel insights into the protective mechanisms of the FLASH effect in reproductive tissues.

## 6 Conclusion

The FLASH effect has been validated in various *in vivo* rodent models across multiple organs, including the skin, heart, lungs, brain, intestines, and spleen. As the central organ of the male reproductive system, the testes play a pivotal role in maintaining fertility. Within the testes, spermatogonia are responsible for the continuous production of sperm. Sertoli cells provide structural and nutritional support for spermatogenesis, while Leydig cells secrete testosterone, regulating male secondary sexual characteristics and reproductive function. Current studies indicate that, under varying dose rates below 40 Gy/s, different cell types within the testes exhibit significantly distinct responses to radiation exposure. However, the hypothesis that testicular tissue also exhibits the FLASH effect is currently based on extrapolations from FLASH studies in other organs and from non-FLASH dose rate studies involving testicular cells. Accordingly, direct experimental evidence remains to be systematically investigated and confirmed. Notably, emerging findings on potential mechanisms of the FLASH effect, including oxygen depletion, free radical dynamics, changes in DNA damage profiles, and immunomodulation, may be intrinsically linked to the structural and functional characteristics of testicular tissue. Therefore, future studies should employ a comprehensive approach—integrating both *in vitro* and *in vivo* experiments using appropriate rodent models, in combination with quantitative physical-biological modeling—to systematically investigate the presence and underlying mechanisms of the FLASH effect in the testes. Furthermore, even under identical FLASH dose rates, physical parameters—including pulse dose, pulse number, pulse interval, pulse width, and total irradiation time—may significantly influence biological outcomes (89). Elucidating the roles of these key mechanisms and parameters in regulating biological responses will not only enhance the potential of the FLASH effect in protecting normal tissues during radiotherapy but also lay a solid foundation for the clinical optimization and broader application of FLASH-RT.
